# 

**Published:** 1835

**Authors:** 


### CONTENTS OF NO. XXXIII.

### ESSAYS AND CASES.

Art. I.—Cholera Fantasies. By the Editor, p. 13
II.—Manual Strangulation. By S. D. Gross, M. D. 25
III.—Siamese Connection Successfully Dissevered. By G. W. Little, M. D. 38
IV.—Hysteria. By Charles Woodward, M. D. 41
V.—Strangulated Hernia. By C. R. Cooper, M. D. 53

### REVIEWS AND BIBLIOGRAPHICAL NOTICES.

VI.—Lesions of the Blood. By M. Andral, 57
VII.—Medical Society of Tennessee, 77
VIII.—Partial Human Combustion. By J. Overton, M. D. 78
IX.—Anatomical Characters of Asiatic Cholera. By W. E. Horner, M. D. 81

### MISCELLANEOUS INTELLIGENCE.

ANALECTIC.

1. A Treatise on Wounds inflicted by the arms of War, by M. Dupuytren, p. 97.—2. On Scrofula, by James Eager, p. 106.—3. Nitrate of Silver in Epidemic Cholera, p. 109.—4. On Manual Assistance in Abortion, p. 110.—5. Observations on Catarrhal and Catarrho-Rheumatic Ophthalmia, p. 114.—6. Lepra Vulgaris invariably treated with success, p. 120.—7. Ophthalmia treated with lotions of Corrosive Sublimate, p. 122.—8. Best mode of treating Mammary Abscess, p. ib.—9. Observations on Erysipelas, p. 124.—10. Anatomico-Pathological Researches on the Pneumo-Gartric Nerve, p. 129.—11. Large Doses of the Tartrate of Antimony after severe wounds, etc. p. 130.—12. Endermic use of Digitalis in Dropsey, p. 131.—13. Detection of the matter of Miasmata in the Air, p. 133.—14. Cases of Fracture of the Carpal Extremity of the Radius, etc. p. 134.—15. Prof. Albers on Inflammation of the Spinal Dura mater, p. 137.—16. On the Treatment of Syphilis without Mercury, p. 139.—17. Memoir of M. Dupuytren, p. 146.

ORIGINAL.

1. Editorial and Publishing Arrangements, p. 154.—2. Medical Schools, p. ib.—3. Western Academy of Natural Sciences, p. 155.—4. Benefits of Practical Phrenology, p. 156.—5. Death of Leaves from the fumes of Lead, p. 157.—6. Nitrate of Silver in Tonsilitis, p. ib.—7. Chalybeates in Dropsy, p. 158.—8. Greenville Epsom Springs, Harrodsburgh, Ky. p. 159.—9. Blue Sulphur Springs, Virginia, p. ib.—10. Exemption of certain parts of the Delta of the Mississippi, from Autumnal Diseases, p. 160.—11. Winter Retreat for those inclined to Pulmonary Consumption, p. 161.—12. Method of finding the Dew-Point, p. 162.—13. Mercurial Transudation, p. 163.—14. Hot Douching in Sprains and Bruises, p. ib.—15. Lithotomy, p. 164.—16. Call for Physicians from the Sandwich Islands, p. 165.—17. Medical Obituary, p. 168.

### SUPPLEMENT TO THE JOURNAL.

1. Reform of the Medical College of Ohio, p. 169.—2. Origin of a Second School, p. 190.—3. Cincinnati College, (Medical Department,) p. 193.—4. To the Friends of Cincinnati Medical Reform in Ohio and the West generally, p. 197.

CONTENTS.

### NO. XXXIV.

### ESSAYS AND CASES.

| | |
|---|---|
| Art. I.—Tumors of the Face. By H. G. Jameson, M. D. | p. 205 |
| II.—Unskillful Surgery. By the same, | 212 |
| III.—Lithotomy and Wounded Rectum. By the same, | 216 |
| IV.—Lithotomy. By Joseph N. McDowell, M. D. | 224 |
| V.—Injury of the Brain. By Dr. P. J. Buckner, | 237 |
| VI.—The Sick Stomach. By the Editor, | 243 |

### REVIEWS AND BIBLIOGRAPHICAL NOTICES.

| | |
|---|---|
| VII.—Materia Medica and Therapeutics. By A. G. Thompson, M. D. | 248 |
| VIII.—Essays and Lectures on Medical subjects. By J. P. Harrison, M. D. | 266 |
| IX.—The Anatomy, Physiology, and Diseases of the Bones. By S. D. Gross, M. D. | 278 |
| X.—A Discourse on Self Limited Diseases. By J. Bigelow, M. D. | 285 |

### MISCELLANEOUS INTELLIGENCE.

### ANALECTIC.

1. Sheet-lead Dressings in Wounds and Ulcers, p. 289.—2. Experimental Trials on the Efficacy of Repeated Purgatives in Typhus Fever, p. 294.—3. Cure of Ascites by Stimulating Injections, p. 296.—4. Hypertrophy of the Muscular Coat of the Stomach, p. 298.—5. Existence of Charcoal in the Lungs, ib.—6. Case illustrating the anomalus nervous symptoms occasionally induced by Tenia, p. 299.—7. On the organization and Origin of Intestinal Worms, p. 301.—8. Pathology of Intestinal Worms, p. 305.—9. On the supposed power of Worms of perforating the intestines, ib.—10. Worms which inhabit the Intestinal Canal in Man, p. 307.—11. Symptoms of Intestinal Worms, 308.—12. Causes of Worms, p. 311.—13. Treatment of Worms, p. 312.—14. Diagnosis of Inflammation of the Spinal Dura Mater, p. 315.—15. Influence of Professions on Pulmonary Consumption, p. 316.—16. On the Probable Duration of Life among Medical Practitioners, p. 319.—18. Method of obtaining the separation of a Necrosed Sequestrum without an operation, p. 323.—19. Nux Vomica in Spasmodic Asthma, p. 324.—20. New method of obliterating Aneurismal Tumors of the extremities, ib.—21. Lepra Vulgaris, ib.—22. Remedy for the Itch, ib.—23. Cure of Jaundice, ib.—24. Newly discovered arrangement of the arteries in the Erectile Tissue of the Penis, p. 325.—25. Abnormal secretion of milk from the Scrotum, p. 327.—26. Remarks on the use of oiled silk and warm water dressing, p. 328.—27. Treatment of Recto Vaginal Communication, ib.—28. Camphorated Citrine Ointment in Hydrocele, p. 329.—29. Catechu in Mercurial Pytalism, ib.

### ANALYTICAL.

1. Diabetes Mellitus cured by Tincture of Cantharides, p. 331.—2. Ligature of the Brachial Artery for the Aneurism from blood-letting, ib.—3. On the mode in which Coichicum operates in the cure of Acute Rheumatism, p. 333.—4. Practical and Pharmaceutic remarks on the Veratrum Viride, p. 335.—5. Case of Extirpation of the Thyroid Gland, p. 338.—6. Researches on the Symptoms and Diagnosis of Aneurism of the Thoracic Aorta, p. 344.—7. Evidence of members of the Royal College of Surgeons, before the British Parliament, p. 353.—8. Physiology of Respiration and chemistry of the blood applied to Epidemic Cholera, p. 361.

### ORIGINAL.

1. Absorption of the Liquor Amnii—Descent of the Gravid Uterus, p. 366.—2. Death following the operation for Congenital Cataract, p. 368.—3. Comparative pain and Constitutional Affections from couching and extracting the Cataract, ib.—3. Safety and success of extracting the Cataract in old age, p. 369.—4. Willoughby and Louisiana Medical Schools, ib.—5. Dr. Jullius of Prussia, ib. —6. Study of General and Pathological Anatomy, p. 370.—7. Analysis of Minerals and Mineral Waters, p. 371.—9. Professor Jameson, ib.—10. Intermittent in the West, p. 372.—11. Medical Observations on certain parts of the Delta of the Mississippi, p. 374.—12. Geological Survey, p. 376.

### NO. XXXV.

### ESSAYS AND CASES.

Art. I.—Fistula of the Urethra. By Dr. R. Estep, p. 377
II.—Wound of the Heart. By R. P. Simmons, M. D. 382
III.—Lesions of Sensations and Volition. By T. N. Maddox, M. D. 339
IV.—Club-Foot. By a Correspondent, 389
V.—Miasm and Contagion. By J. L. Riddell, M. D. 401

### REVIEWS AND BIBLIOGRAPHICAL NOTICES.

VI.—Monstrosities. By M. Andral, 413
VII.—Materia Medica. By A. G. Thompson, M. D. 426
VIII.—Medical Cyclopedia. —— —— 444
IX.—Pulmonary Consumption. By James Clark, M. D. 451

### MISCELLANEOUS INTELLIGENCE.

ANALECTIC.

1. Diuretic Medication, p. 460.—2. Therapeutic employment of Vapor Baths in certain Ocular Affections, p. 462.—3. Therapeutics and Contractions of the Uretha, p. 463.—4. New facts relative to the action of Cresote, ib.—5. Of the action of Tannin on the Salafiable Bases, ib.—6. Experiments on the connexion of the Nervous System and the Irritability of Muscles in Living Animals, p. 464.—7. Vital Properties of Arteries leading to Inflamed Parts, by Dr. Allison, p. 466.—8. Experimental Inquiry regarding the sensibilities of the Cerebra Nerves, by M. Hall and Mr. Broughton, p. 468.—Facial Nerves, p. 469.—Nerves Nagus—Spinal Accessary—Glossol Pharyngeal—Ninth Pair and Great Sympathetic, p. 470.—9. Serpentine direction of the Arteries in different Animals, p. 472.—10. Pathology of Apoplexy, by Wm. Stokes, M. D., p. 473.—11. Circumstances which predispose to Apoplexy, by Wm. Stokes, M. D., p. 479.—12. Diagnosis of Apoplexy, by Wm. Stokes, M. D., p. 480.—13. Treatment of Apoplexy, by Wm. Stokes, M. D., p. 486.—14. Paralysis consequent on Apoplexy, by Wm. Stokes, M. D., p. 488.—15. Paralysis from Disease of the Arterial System, by Wm. Stokes, M. D., p. 490.—16. Diagnosis of Paralysys from Disease of the Arterial System, by Wm. Stokes, M. D., p. 493.—17. Paraphlegia, by Wm. Stokes, M. D., p. 594.—18. Treatment of Paralysis consequent on Apoplexy, by Wm. Stokes, M. D., p. 500.—19. Epilepsy cured by Indigo, p. 506.—20. Amenorrhœ cured by Turpentine Injections, ib.—21. Breschet's operation for the radical cure of Varicocele, p. 507.—22. Simple Fracture of the Os Femoris—Reunion—Death and Dissection, by Jas. Symes, p. 509.—23. Prolapsus Ani, cured by Nux Vomica, p. 510.—24. Notice of a case of Urinary Calculus, in which Dr. Randolph successfully performed Lithotripsy, by J. Hays, M. D., 511.

ANALYTICAL.

1. The late Dr. David Hosack, p. 513.—2. Radical cure for Hernia, p. 515.—3. Call of the Maryland Academy of Science and Literature, p. 519.—4. Diet, p. 520.

CONTENTS.

ORIGINAL.

1. Our Periodical, p. 521.—2. Medical Department of Cincinnati College, p. 524.—3. Tribute to Professor McDowell, p. 526.—4. Conclusion of Miasm and Contagion, p. 526.—5. Note from the Publishers, p. 532.

### CONTENTS OF NO. XXXVI.

ESSAYS AND CASES.

ART. I.—Relations between the food and flesh of animals, p. 555
II.—Meteorology. By Prof. Ray. 547
III.—Supplementary Catalogue of plants. By J. L. Riddell, M. D. 567
IV.—Hydrophobia. By O. M. Herron, M. D. 592

REVIEWS AND BIBLIOGRAPHICAL NOTICES.

V.—Physiology. By P. M. Roget. 599
VI.—Western Medical Schools. 608
VII.—Journal of Pharmacy. 618

MISCELLANEOUS INTELLIGENCE.

ANALECTIC.

I. Treatment of Paralysis from diseases of Arterial System, by Wm. Stokes, M. D. p. 647.—2. Employment of Blood-letting, in Scarlet Fever, p. 647.—3. Sulphate of Copper, a remedy for Croup, p. 652.—4. Changes produced in the composition of the Blood, by repeated blood-letting, p. 653.—5. Researches on the Blood, by Gmelin and Tiedemann, p. 656.—6. Physiological and Chemical researches on the blood of the Vena Porta, p. 659.—7. Case illustrative of the Ophthalmic Branch, of the 5th pair of nerves on the nutrition of the Eye, p. 660.—8. Observations on malignant Pustule, p. 662.—9. Remarks on Cough, by Robt. Graves, M. D. p. 666.—10. Notice of a new mode of preserving bodies, p. 673.—11. Solidification of Turpentine by Magnesia, p. 675.—12. Diabetes cured, p. 676.—13. On Tic Doloreux, by Wm. Stokes, M. D. p. 676.—14. Digitalis, a specific for Delirium Tremens, p. 678.—15. Pulmonary Hæmorrhage, treated with Ergot, p. 678.

ORIGINAL.

I. Medical History of the West, p. 680.—II. Medical Department of Cincinnati College—Edifice—Trustees, p. 685.—III. Mnemonic Nomenclature, p. 687.—IV.—Metastasis of Disease from the Bowels to the Brain, p. 691.—V. Gangrene of the Lungs following Small-pox, p. 693.

